# Impacts of climate change to African indigenous communities and examples of adaptation responses

**DOI:** 10.1038/s41467-021-26540-0

**Published:** 2021-10-28

**Authors:** Walter Leal Filho, Newton R. Matandirotya, Johannes M. Lütz, Esubalew Abate Alemu, Francis Q. Brearley, Anastasia Ago Baidoo, Adolphine Kateka, George M. Ogendi, Girma Berhe Adane, Nega Emiru, Richard Achia Mbih

**Affiliations:** 1 Department of Natural Sciences, Manchester Metropolitan University, Chester Street, Manchester, M1 5GD UK; 2 Research and Transfer Centre “Sustainable Development and Climate Change Management”, Hamburg University of Applied Sciences, Ulmenliet 20, D-21033 Hamburg, Germany; 3 Unit for Environmental Sciences and Management, North-West University, Private Bag X6001, Potchefstroom, 2520 South Africa; 4 Kgotso Development Trust, P.O. Box 5, Beitbridge, Zimbabwe; 5 School of Social Sciences, Christian Heritage College, Brisbane, QLD Australia; 6 School of Social Sciences, University of New South Wales, Sydney, NSW Australia; 7 School of Law and Society, University of the Sunshine Coast, Sippy Downs, QLD Australia; 8 Centre for Rural Development, College of Development Studies, Addis Ababa University, Addis Ababa, Ethiopia; 9 Tanzania Water Partnership, P.O. Box 14200, Dar es Salaam, Tanzania; 10 Ecotourism Centre, Egerton University, P.O. Box 439, Egerton, 20115 Kenya; 11 School of Water Resources and Environmental Engineering, Haramaya University, Institute of Technology, P.O. Box 138, Dire Dawa, Ethiopia; 12 Ethiopia Agricultural Transformation Agency, Addis Ababa, Ethiopia; 13 The Pennsylvania State University, African Studies Program, University Park, PA 16802 USA

**Keywords:** Research management, Governance

## Abstract

Climate change negatively impacts the livelihoods of indigenous communities across the world, including those located on the African continent. This Comment reports on how five African indigenous communities have been impacted by climate change and the adopted adaptation mechanisms.

## Local knowledge use for climate-change adaptation by African indigenous communities

Globally, there are an estimated 370 million indigenous people^1^ whose livelihoods are being negatively affected by climate change^2^ by means of an increased frequency and intensity of extreme weather events such as droughts, floods, storms, cyclones, as well as heatwaves, among others^1^. While climate change is an environmental challenge that developed countries have largely contributed toward from anthropogenic activities, the negative impacts are being felt among poorer countries, particularly vulnerable indigenous communities who ordinarily live low carbon lifestyles^1,3^. Additionally, many indigenous communities have been confined to the least productive and most delicate lands because of historical, social, political, and economic exclusion^4^. Furthermore, less consideration has been given to indigenous groups during formulation of climate-change mitigation strategies, making them vulnerable to its effects^5^. Notwithstanding, many indigenous communities have enduringly used various indigenous and local knowledge (ILK)-derived coping mechanisms passed from generation to generation.

Here, we provide examples of the various climate-change-related challenges faced by five African indigenous communities (Afar, Borana, Endorois, Fulani, and Hadza) and the various adaptation mechanisms they use. After examining African indigenous communities in the context of international trends, we offer a broader outline of the role indigenous communities can play in combating climate change by conserving environmental resources in their lands and territories, including a description of future trends and suggestions of measures via which the vulnerability of indigenous communities may be reduced.

Over recent years, scholars have drawn attention to the underappreciated potential of ILK to help manage and redress food insecurity in the face of climate change^6^, including Africa^7^. Moreover, scholars have called for so-called reversals of learning whereby indigenous communities, which are sometimes denigrated simply as being poor, rather “teach the profligate and so-called ‘developed’ rich about the interwoven nature of frugality, modesty, contentedness, spirituality and sustainability”^8^. There is, therefore, a compelling case to engage closely with ILK so that it may more effectively inform the global climate-change adaptation agenda. Understanding the strategies derived from ILK that indigenous groups have used to deal with ecological uncertainty (a.k.a. environmental risk) such as droughts, food insecurity, and loss of, or displacement from land, and how they build resilience against climate-related stresses and shocks, can be harnessed for present and future community application. Resilience refers to the capacity of a system to anticipate, reduce, accommodate, or recover from the effects of a hazardous event^9,10^, however, the resilience of indigenous communities under new climate-change-induced shocks is under pressure. Importantly, different indigenous groups can exhibit different degrees of resilience to different stresses due to the socioeconomic, demographic, cultural, and political factors at play that^11^ have grouped under a framework of place, agency, institutions, and collective action. Even so, under current trends, the rates and variabilities of contemporary climate changes threaten to outpace and overwhelm the adaptive capacities of many indigenous societies^12^.

## Trends in adaptation to climate change among African indigenous communities

Indigenous communities have been constantly adapting to the effects of environmental stresses over a very long period with numerous climate-change adaptation mechanisms being adopted in recent decades. However, more recent impacts of climate change have placed significant strain on these communities^13^ as indigenous people are impacted in idiosyncratic ways by climate change (e.g., reduction in crop yields, water scarcity, and exposure to malnutrition) and also by the failed policies or actions that are designed at addressing it.

**Fig. 1:**
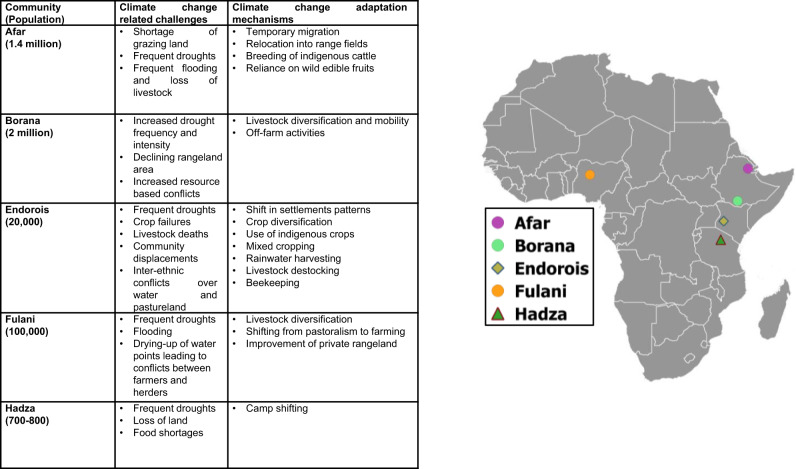
Spatial distribution of different African communities, climate-change-related challenges faced, and adaptation mechanisms being applied.

Figure 1 provides an overview of the climate pressures these communities spread across Africa are currently exposed to, and the adaptation mechanisms they are deploying, so as to be in a better position to cope with the challenges posed by a changing climate.

Apart from the matters described in Fig. 1, there are further barriers to climate change adaptation that are often seen across Africa, namely the unequal global vulnerability of populations, differential responsibility, and unequal power in decision-making concerning policymaking, thus undermining the resilience capability of indigenous communities^14^. As seen in Fig. 1, the studied communities are making their best efforts to address these barriers. There are examples that show that indigenous people’s knowledge is one important component to the success of policies that aim to increase adaptation^15^. For instance, the Afar communities have extensive experience in adapting to the impacts of climate change using their ILK via understanding the biophysical observations^16^, and the community’s perception was matched with the temperature trends using conventional weather-forecasting systems^17^. Similarly, the Borana people have been using indigenous collective resource-governance systems, traditional social insurance and safety-net systems, and weather-forecasting systems based on changes in animal behaviors, as well as the movement and alignment of stars and divining animal entrails, which have proven to be reliable for centuries despite the challenges posed by an increasingly variable climate^18^, thus allowing acclimatization to drought challenges^19^.

The Fulani people have also used such indigenous climate-adaptation techniques, such as livestock-feed diversification, cattle stress-management techniques, and division of labor^20^. On the other hand, the Endorois people have turned to climate-smart agroecological production systems such as the cultivation of drought-tolerant cereals, tubers, and vegetables. This shift in production systems has led to more sustainable land management, minimized water usage, reduced human–wildlife conflict, and enhanced food security among the Endorois^21^. Owing to their close cultural connection to their environment, the Endorois have also embraced nature-based ecotourism enterprises, including medical, cultural, and geotourism in response to the climate-change-induced negative effects on the livelihoods. Other adaptations to climate-change effects among the Endorois people include livestock and crop diversification, herd adjustment by class, livestock destocking, and supplementary feeding of livestock^21^.

It is a matter of fact that ILK for climate-change adaptation is not limited to African communities. In Australia, the Mirriwong people, for example, have adopted the use of fauna and flora as an instrument of monitoring seasonal changes, for example, the flowering of Woolegalegeng (*Melaleuca argentea*) signals thunderstorms^22^. On the other hand, in Malaysia, the communities of Sarawak (Lun Bawang, Saban, and Penan) have used indigenous forecasts such as sky-color changes, moon phases, and animal migration^23^ to identify changes in weather patterns. International examples highlight the potential for ILK systems to be integrated into modern climate-risk assessments as part of adaptation to climate-change-related hazards^18,20^.

## Challenges and opportunities for the future of indigenous people’s climate adaptation

In light of the adverse effects of climate change, indigenous people in Africa face some challenges, which need to be addressed. For instance, these groups are often hard-pressed to maintain their unique land-use and tenure systems, which are being degraded by unfavorable climatic conditions. Also, because the livelihoods of the indigenous communities and minority groups in Africa are closely associated with their environment, there are numerous climate-change-related impacts that pose a threat to their well-being, especially nutrition.

The first part of the 6th Assessment Report issued by the Intergovernmental Panel on Climate Change^24^ has indicated that increases in both the frequency and intensity of extreme events may be expected. This trend suggests that indigenous communities may be under additional pressure to handle unfavorable climate conditions and are trying to adapt to climate change through different mechanisms.

As this Comment has illustrated, African indigenous communities are trying to adapt to the changes through different mechanisms. In contrast, the governing framework at international, national, and regional levels in response to the negative impacts of climate change does not effectively protect indigenous people’s interests, including their culturally valued lifestyles, livelihoods, and resources—which is particularly concerning, given that indigenous people have not contributed to climate change in any significant way. This paradox illustrates the need for a human-rights framework implementation at the local level, to help them to address the challenges they currently face^23^. For climate action to be fruitful, indigenous people also need to be seen as prime agents of change. While contemporary climate-change adaptation efforts overwhelmingly privilege Western scientific knowledge and technocratic management approaches, there is a danger of side-lining and marginalizing ILK as negligible or insignificant, leading to a failure to take into account indigenous knowledge in implementing climate action^12^.

At the same time, it is also important that the drivers (e.g., droughts), which increase the exposure of indigenous people to climate change, are addressed in a distinctive and targeted manner^15^. Many tribes have robust local agroecological knowledge and are naturally engaged in climate-change adaptation and mitigation strategies, but some tribes/communities have limited knowledge on the development of climate-change adaptation strategies. Additionally, there is limited access to resources and technology that are required to implement specific types of adaptation^3^.

Traditional and local ecological knowledge of indigenous people can help to bolster food security and allow for sustainable management of ecosystems that, in turn, may mitigate the effects of climate change^25^. However, there are now clear signs that ILK is under threat of being side-lined, marginalized or even lost^26^. There are strong synergies to be obtained between strategies that promote socioecological resilience, climate-change mitigation and livelihood benefits to indigenous groups.

Indigenous and local knowledge has been, and shall continue to be, deployed by indigenous communities as part of coping strategies for protecting assets and livelihoods and adapting to climate change. The evidence presented in this Comment highlights the need for national governments, international organizations, and other stakeholders to give due attention and support to the use (and documentation) of traditional and local knowledge, so that indigenous communities may better withstand the negative impacts of climate change.
